# Hugh Esmor Huxley (1924–2013)

**DOI:** 10.1007/s10974-013-9365-6

**Published:** 2013-11-14

**Authors:** Kenneth Holmes

**Affiliations:** Max Planck Institut für medizinische Forschung, Heidelberg, Germany

Hugh Esmor Huxley was born on 25th February 1924 and raised in Birkenhead, Cheshire across the Mersey river from Liverpool. His family originated in North Wales and were generally of a schoolmasterly persuasion. Hugh attended the Park High School where he became school captain. He spent much of his adolescence making short wave radio receivers. He was also a keen experimentalist: his most ambitious experiment was an attempt to make diamonds by dissolving carbon in molten metals in a home-made electric furnace. Both he and his 7-year elder sister gained admittance to Cambridge University, he to Christ’s College in 1941. Hugh was fascinated by quantum physics and the possibilities of nuclear power saving mankind from an energy crisis. He completed his Part I unusually quickly and was offered the chance of starting his Part II in Physics in his second year. But Britain was at war; Hugh elected to interrupt his studies and to join the Royal Air Force working on the development of radar. He was honoured for his substantial contributions to this very important field by being awarded membership of the Order of the British Empire (MBE).

He returned to Cambridge in 1947, his enthusiasm for Nuclear Physics irrevocably diminished by the horrors of Hiroshima. Notwithstanding his moral reservations about physics he achieved a first class degree and elected to do his doctoral research at the Cavendish Laboratory. Rather than nuclear physics he sought out Max Perutz’s small group working on the X-ray analysis of crystalline proteins, where he became John Kendrew’s first research student. John entrusted Hugh with calculating the Patterson function of diffraction data from a sheep hemoglobin crystal. Hugh felt this tedious job was unbefitting the human condition and persuaded John that he might do something else. Hugh had come across Dick Bear’s X-ray diffraction patterns from air-dried muscle and, moreover, was amazed to discover no one knew how muscle works. The tradition of the Perutz group was to keep things wet, so Hugh set about building an X-ray camera and X-ray generator capable of resolving spacing of 300–400 Å to take diffraction photographs of wet “living” muscle to see what you could see. He saw for the first time the equatorial reflections arising from a hexagonal array of filaments and somewhat prophetically guessed that these arose from filaments of myosin on the hexagonal lattice points with actin filaments in between. The idea of actin and myosin being organized in distinct filaments was quite new and stems from Hugh. He also suggested the filaments were linked by “cross bridges”. He noted the large change in relative intensities when the muscle goes into rigor. He also noted that the meridional reflections don’t alter as the muscle is stretched, which argued strongly against the filaments bringing about contraction by altering their structure in some way—the then current explanation of contraction.

Hugh’s Ph.D. viva in June 1952 was with Sir Lawrence Bragg, the Cavendish Professor, and Dorothy Hodgkin from Oxford. Dorothy was intrigued by Hugh’s report of the strong changes in the equatorial reflections that occur when a muscle goes into rigor. She assumed (incorrectly) that the muscle shortens as it goes into rigor and intuited (correctly) that the changes of intensities Hugh reported would be produced by letting two sets of interdigitating filaments slide to produce more overlap. According to Hugh she illustrated her idea by interdigitating the fingers of her left and right hands to represent two sets of filaments and then let them slide together. Hugh pointed out that he had gone to a lot of trouble to make sure that the filaments stayed at constant length when he let them go into rigor and with some irritation dismissed Dorothy’s sliding filament hypothesis!

In 1952 Hugh went to the Massachusetts Institute of Technology (MIT) with a Commonwealth Fellowship as a post-doc in Frank Schmitt’s department to learn about electron microscopy. Soon his transverse sections of fixed muscle showed the arrangement of filaments he had postulated from his X-ray studies together with a hint of cross-bridges connecting the filaments. However, it was the arrival of Jean Hanson at MIT (also intent on learning electron microscopy) that provided the impetus for understanding sliding filaments. Jean was experienced in the then new technique of phase contrast microscopy. The units of cross-striated muscle are sarcomeres, about 2.5 μ in length, delineated by Z-lines, all in series with each other. In the phase contrast pictures of single muscle fibers the sub-divisions of the sarcomere into H-zone, A-band and I-band can be seen easily. Examining glycerinated rabbit muscle fibers after extraction of the myosin with Hasselbach–Schneider solution, which dissolves myosin, it became clear that filaments in the I-bands were just actin, the H-zones were just myosin, and the A-bands were overlap zones for actin and myosin filaments. Carrying out these experiments at various degrees of stretch of the muscle demonstrated that the muscle consists of two sets of filaments that move past each other as muscle contracts. Hugh and Jean wrote up the results for Nature but on the advice of Frank Schmitt left out the sliding filament hypothesis: he maintained *good data should not be spoiled with speculation*. This may have been bad advice since this ground-breaking work was never acknowledged by the Nobel committees. Nevertheless, the idea that myosin was confined to the A-band was already enough to upset the establishment. Albert Szent Györgyi, now in Woods Hole, was not amused. He knew how muscles contract. Myosin is a negatively charged polymer: when you add calcium ions it undergoes a phase change and shortens. However, if myosin doesn’t extend all the way between the Z-lines this can’t work!

While in Boston Hugh formed a life-long friendship with Andrew Szent Györgyi, Albert’s younger cousin. Hugh often went down to the Marine Biological Laboratory in Woods Hole during summer weekends, and stayed with Andrew and Eve Szent-Györgyi. In the 1950s Andrew made an important advance in myosin biochemistry by preparing “light meromyosin” (LMM) and “heavy meromyosin” (HMM)—proteolytic fragments of myosin. HMM was soluble in low salt. It contained the ATPase and was basically a pair of cross-bridges. Now myosin biochemistry could move forward.

In the summer of 1953 Hugh met his namesake (but not relative) Andrew Huxley in Woods Hole. Unlike the muscle establishment Andrew was sympathetic to Hugh’s ideas of sliding filaments and indeed, using his own sophisticated interference microscope, was reaching similar conclusions. The next year, together with Jean Hanson and Rolf Niedergerke they published the sliding filament hypothesis in two papers back-to-back in Nature. This was the beginning of modern muscle research.

In the Spring 1954 Hugh returned to Cambridge, England, to a research fellowship at Christ’s College but in 1956 he moved to a position at University College, London, in Bernard Katz’s Department where the Wellcome Foundation provided him with a new Siemens Elmiscope electron microscope. Here he made substantial advances in the fixing and sectioning of muscle. He built a microtome capable of cutting ultra-thin sections. He produced wonderful images of longitudinal sections of cross-striated muscle barely 15.0 nm thick clearly showing the “cross-bridges” connecting the thick (myosin) and thin (actin) filaments. In collaboration with Bernard Katz he produced stained images of the neuromuscular junction. Further, he and Geoffrey Zubay developed “negative staining”: if you immerse a biological macromolecule in a solution of a heavy metal salt on a microscope grid and let it dry to form a glass, the electron micrograph shows a cast of the macromolecule in the heavy metal glass. This method found wide application and became very important in unraveling virus structure. Using negative staining Hugh discovered that if one applied Andrew Szent Györgyi’s HMM preparation to actin filaments the cross-bridges bound specifically and regularly to the actin filament, one cross-bridge per actin, with the symmetry of the actin filament. Immediately this allowed Hugh to see, with some wonderment, that the polarity of the actin filaments reverses as you go through the Z-line, which is actually essential if sarcomeres are to shorten in series with each other. This construct became known as “decorated actin” and was often used for establishing the polarity of actin filaments in cell biological preparations. Ten years later it was the first object with helical symmetry to be reconstructed in three dimensions by the method developed by Aaron Klug and David DeRosier. Finally, some 50 years later, high resolution electron microscopy of decorated actin allowed one to see in detail how myosin binds to actin thus finally enabling an understanding of muscle contraction at the atomic level.

In 1962 Hugh moved back to Cambridge, to the newly opened MRC Laboratory of Molecular Biology (LMB) and to a research Fellowship at Kings College. His corner set in Bodley’s Court overlooked the Cam and became a venue for biweekly classical music recitals with Hugh’s state of the art Hi-Fi system. At the LMB he set up a Siemens Elmiscope-1 and used it to investigate the spontaneous assembly of myosin molecules in vitro to form bipolar thick filaments, again an essential requirement for sarcomeric shortening. He taught John Finch how to take negative stain images of viruses, which contributed substantially to Aaron Klug’s Nobel winning work on virus structure.

In February 1966 Hugh married Frances Maxon Fripp from Boston. They had met at Duke University Medical School while visiting J.D. Robertson. Frances brought three teenage children into the marriage. They duly moved to Cambridge, England. The family initially lived at “Binstead” a lovely house on Herschel Road. Binstead, which belonged to St. Johns College, was later torn down to make room for Robinson College—only the Bin brook remains. In December 1970 Frances gave birth to Olwen. The Huxleys later moved to Chaucer Road. The Fripp children slowly drifted back to Boston.

Hugh was still intrigued by the low angle X-ray diffraction pattern of muscle that he had uncovered in his doctoral research. Using much better X-ray sources and optical systems developed at the LMB he was now able to follow in some detail the changes in the X-ray patterns when a muscle goes into rigor. The next challenge was to follow the changes when a resting muscle is stimulated. However, the experiments were still difficult and one still didn’t have enough X-ray intensity to see what happened during a contraction. According to the “swinging cross-bridge” hypothesis that he first presented in a Scientific American article in 1958 and more formally as a review in Science in 1969, the cross-bridges bind in an initial conformation and “row” the actin filaments past the myosin filaments by going over into a second more angled conformation followed by release from actin and rebinding in the first conformation. In 1971 Ed Taylor and Richard Lymn elegantly connected the swinging cross bridge hypothesis with the binding and hydrolysis of ATP in the “cross-bridge cycle”. By rapid recording of length and tension Andrew Huxley and Bob Simmons were able to show that when an actively contracting muscle is released the first elastic response is followed by a quick recovery produced by active cross-bridges moving about 10.0 nm. Thus the swinging cross-bridge hypothesis appeared to be fully consistent with known structural, biochemical and physiological data. Nevertheless, for many years the swinging cross-bridge remained not much more than an elegant idea because, apart from muscle tension, no signals were available to show that something was happening. Hugh knew that a change in orientation of the cross-bridge would manifest itself as a change in the low angle X-ray scattering pattern, if you could get enough intensity to record the diffraction pattern from an stimulated frog muscle in a couple of milliseconds. Sadly, conventional X-ray sources had reached their limit. Thus Hugh was enthusiastic about my experiments at the DESY synchrotron in Hamburg in 1970 showing that synchrotron radiation from an electron synchrotron or storage ring was a potential X-ray source for diffraction experiments of almost unlimited intensity. With Hugh’s support the newly founded European Molecular Biology Laboratory in Heidelberg, where Hugh’s ex-doctoral supervisor John Kendrew was Director, was persuaded to open an outstation at DESY Hamburg for the exploitation of synchrotron radiation as an X-ray source. Here in 1981 Hugh and his team of collaborators were finally able to demonstrate that swinging cross-bridges actually swung.

The low angle X-ray diffraction of contracting muscle shows a relatively strong reflection on the meridian of the X-ray pattern (i.e. in the direction parallel to the axis of the muscle) at a spacing of 14.5 nm. Apparently as an act of benevolence to muscle physiologists, this reflection arises solely from myosin cross-bridges attached to actin. If the cross-bridges stick out at right angles to the filament axis, that is near the beginning of the cross-bridge working stroke, this reflection is strong. If they are angled, at the end of the working stroke this reflection is weak. Hugh’s experiments showed that the 14.5 nm reflection gets much weaker during the elastic phase, and recovers either by pulling the muscle out again or waiting a few milliseconds for the cross-bridges to cycle back to their original state.

At the Laboratory of Molecular Biology Hugh investigated the structures of thin filaments. He and John Haselgrove were able to find an X-ray diffraction signal from muscle that showed how tropomyosin, one component of the thin filament, moved in response to activation so as to free up the cross-bridge binding site on actin. Further Hugh and John Haselgrove investigated the structures of myosin filaments, both by electron microscopy and low angle X-ray diffraction. Thick filaments from vertebrate cross-striated muscle undergo a still rather mysterious apparent change of structure when the muscle is activated. Effort was spent developing the methodology for the time-resolved X-ray diffraction experiments to follow quick release of contracting muscle. The first experiments were done on the British synchrotron at Daresbury, Cheshire, some 15 miles from Hugh’s birthplace. Unfortunately the synchrotron was not bright enough. Successful experiments had to wait for the implementation of X-ray beam lines on the DORIS storage ring at DESY Hamburg.

It was becoming clear that for a detailed understanding of the myosin cross-bridge as a molecular machine it would be necessary to determine the atomic structure of the cross-bridge by X-ray crystallography. In 1967 Hugh appointed Alan Weeds to head a biochemistry group working on myosin. Alan had done ground-breaking work in Susan Lowey’s group at the Rosenstiel Center at Brandeis University in producing homogeneous “cross-bridges” known as subfragment-1 (S1) by controlled digestion of myosin with chymotrypsin. It was hoped that homogeneity would allow the S1 to crystallize. This turned out to be very difficult, probably because the myosin cross-bridge can take on multiple configurations. Crystallisation was finally achieved 20 years later by Don Winkelmann and Ivan Rayment at the Rosenstiel Center at Brandeis. By this time Hugh had moved back to Boston and was able to oversee these very important developments as Director of the Rosenstiel Center.

In the mid 1960s Hugh consulted with Herbert (Freddie) Gutfreund, Hugh’s close friend from the early Cambridge times, about the possibility of using fast enzyme kinetics to explore the mechanism of the actin–myosin ATPase. Freddie turned his attention to myosin and started a group in Bristol that made very significant advances in understanding the details of the myosin ATPase. Because of the quality of his chymotryptic subfragment-1 Alan was strongly involved in this work.

In 1968 Jim Spudich came to the LMB Cambridge to work with Hugh on the electron microscopy of decorated actin filaments. This visit stimulated Alan’s interest in actin and actin binding proteins that later became his main research topic. Jim was curious about the function of tropomyosin and troponin in controlling myosin (S1) binding to the thin filament. It was difficult to get actin filaments (f-actin) free from contamination with tropomyosin and troponin. Jim collaborated with Alan’s technician Susan Watt to develop a method of making pure f-actin. The ensuing publication containing the actin preparation procedure became the most cited paper in the actin literature.

**Fig. 1:**
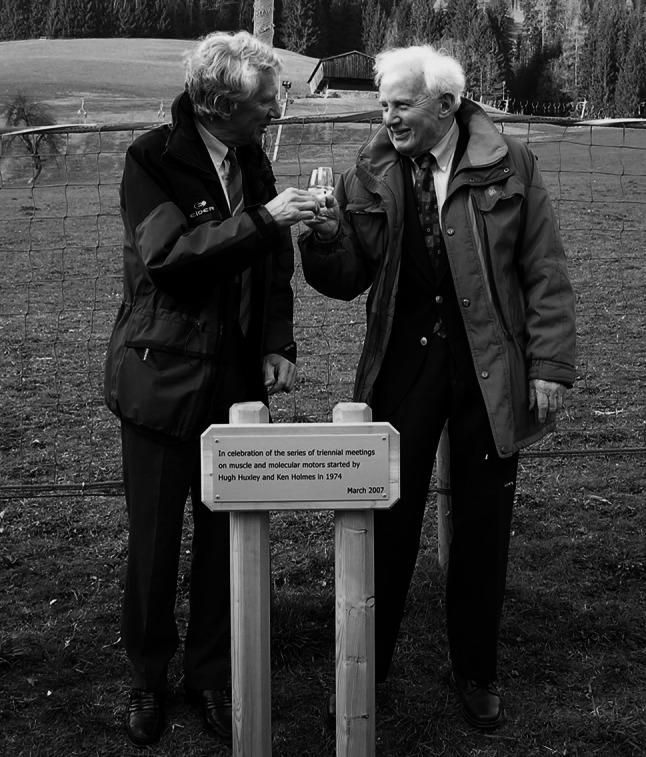
Hugh Huxley and the author in Alpbach, 2007

In 1972 Hugh Huxley and Aaron Klug organized a very successful meeting on electron microscopy at the Royal Society in London. Two days later a meeting on the same topic had been organized by Walter Hoppe in Hirschegg, an Austrian ski resort. Somehow the scientific program had been arranged so that the afternoons were free for discussion in whatever format you felt was appropriate. One passion in Hugh’s life was skiing. Thus at the end of the London meeting Hugh jumped into his splendid blue Jaguar saloon and drove off to Hirschegg. He was impressed by the format of the Hirschegg meeting, which was followed the next year by a similar meeting organized by Max Perutz and Walter Hoppe in the outstandingly beautiful Tirolean village of Alpbach. Hugh and I then resolved to hold muscle meetings in Alpbach at skiing time on a three year cycle to interleave with the Muscle Gordon Conferences. The first of the Alpbach Muscle Meetings, held in 1974, took place in an insalubrious cellar room that was used by the local brass band for rehearsals. Somewhat to our surprise everyone with a name in muscle research turned up. Fumio Oosawa and Setsuro Ebashi came from Japan. Endre (NA) Biro from Budapest chain-smoked at the back. John Pringle came from Oxford. Ed Taylor argued with Evan Eisenberg about myosin ATPase kinetics and Manuel Morales regarded it all with an aristocratic detachment. At the end of the week Andrew Huxley was given the unenviable job of summing up. Andrew really didn’t trust explanations of muscle contraction that contained phrases like “conformational change”. Luckily for Andrew, after the spurt of activity culminating on the sliding filament model of muscle contraction structural explanations dried up. Finally in the last decade of the twentieth century X-ray structure analysis of crystalline myosin in three different conformations did provide an explanation of muscle contraction at atomic resolution but for about 20 years there was a hiatus. In the mean time the series of Alpbach meetings established themselves as a forum for heated discussions of how myosin cross bridges might swing. Hugh could also go skiing (Fig. 1).

Together with Aaron Klug, Hugh became joint head of the Division of Structural Studies. Later he became deputy Director of the LMB but the shadow of impending retirement now loomed over him. The MRC had a policy that scientists should retire at 65. Sidney Brenner, the then director of LMB, was firmly of the opinion that this rule should be enforced. Fortunately, Hugh was offered a professorial appointment at the Rosenstiel Basic Medical Sciences Research Center at Brandeis, which he accepted. In 1987 the family moved to Concord, Massachusetts, within easy reach of Brandeis. In 1988 Hugh accepted the Directorship of the Rosenstiel Center, a position he held for six years. Later he became a professor emeritus and continued funding his research with NIH grants.

Hugh and Frances built a house in Woods Hole where Frances spent her summers directing local drama groups and sailing. On the far side of the Eel Pond lay the sloop “Saraband” that Hugh had imported from England. Hugh came at weekends and spent much of the time tinkering with Saraband or sailing. After Gordon Conferences on muscle Hugh would invite friends and ex post docs to visit Woods Hole to enjoy a not always uneventful trip to Martha’s Vineyard. On the middle ground Hugh would invariably produce his fishing tackle.

In 1997 the Advanced Photon Source, an electron storage ring at the Argonne National Laboratory, became available as the world’s brightest X-ray source. The BIO-CAT team led by Tom Irving set up excellent beam lines for low angle X-ray scattering with fast X-ray detectors. These provided Hugh with the opportunity to investigate a phenomenon first explored with single muscle fibres by Vincenzo Lombardi and his collaborators: the 14.5 nm meridional reflection is actually split into two closely spaced reflections that arise from interference between the two halves of the sarcomere. Because it is an interference phenomenon, careful measurements of the splitting allows the average position of the cross-bridges attached to actin to be measured with high precision. Using intact frog muscles Massimo Reconditi and Hugh were able to work out the distribution of cross-bridges during a contraction. Perhaps surprisingly, during a contraction most of the cross-bridges are near the beginning of their working stroke. This work was reported in two papers in the *Journal of Molecular Biology* that demonstrate Hugh’s characteristic rigor and analytical ability. They are a fitting conclusion to Hugh’s scientific career and provide an elegant vindication of the swinging cross-bridge.

In the last year of his life, as a personal tribute to the Laboratory of Molecular Biology in Cambridge, Hugh edited a volume of 41 reminiscences from visiting scientists, including three Nobel prize winners, to mark the 50 anniversary of the opening of the Laboratory.

Hugh died unexpectedly on 25th July 2013 in Woods Hole. Active to the last, he combined a wonderful experimental ability with a very analytical mind. He was also a gentle humanist and a man of great integrity. His originality and creativity led to his being elected to the Royal Society of London at the young age of 36. He was a member of the National Academy of Sciences. In 1971 Hugh was awarded the Louisa Gross Horwitz Prize. To his delight Columbia University offered two transatlantic flights on the supersonic Concorde. In 1975 he received the Gairdner Award. In 1997 he was awarded the prestigious Royal Society Copley Medal. The citation reads:

In recognition of his pioneering work on the structure of muscle and on the molecular mechanisms of muscle contraction, providing solutions to one of the great problems in physiology

